# The three numbers you need to know about healthcare: the 60-30-10 Challenge

**DOI:** 10.1186/s12916-020-01563-4

**Published:** 2020-05-04

**Authors:** Jeffrey Braithwaite, Paul Glasziou, Johanna Westbrook

**Affiliations:** 1 Centre for Healthcare Resilience and Implementation Science, Australian Institute of Health Innovation, Macquarie University, Level 6, 75 Talavera Road, Sydney, New South Wales 2109 Australia; 2 Institute for Evidence-Based Health Care, Faculty of Health Sciences and Medicine, Bond University, Level 2, Building 5, 14 University Drive, Robina, Queensland 4226 Australia; 3 Centre for Health Systems and Safety Research, Australian Institute of Health Innovation, Macquarie University, Level 6, 75 Talavera Road, Sydney, New South Wales 2109 Australia

**Keywords:** Learning health system, Complexity, Complexity science, Change, Evidence-based care, Clinical networks, Quality of care, Patient safety, Policy, Healthcare systems

## Abstract

**Background:**

Healthcare represents a paradox. While change is everywhere, performance has flatlined: 60% of care on average is in line with evidence- or consensus-based guidelines, 30% is some form of waste or of low value, and 10% is harm. The 60-30-10 Challenge has persisted for three decades.

**Main body:**

Current top-down or chain-logic strategies to address this problem, based essentially on linear models of change and relying on policies, hierarchies, and standardisation, have proven insufficient. Instead, we need to marry ideas drawn from complexity science and continuous improvement with proposals for creating a deep learning health system. This dynamic learning model has the potential to assemble relevant information including patients’ histories, and clinical, patient, laboratory, and cost data for improved decision-making in real time, or close to real time. If we get it right, the learning health system will contribute to care being more evidence-based and less wasteful and harmful. It will need a purpose-designed digital backbone and infrastructure, apply artificial intelligence to support diagnosis and treatment options, harness genomic and other new data types, and create informed discussions of options between patients, families, and clinicians. While there will be many variants of the model, learning health systems will need to spread, and be encouraged to do so, principally through diffusion of innovation models and local adaptations.

**Conclusion:**

Deep learning systems can enable us to better exploit expanding health datasets including traditional and newer forms of big and smaller-scale data, e.g. genomics and cost information, and incorporate patient preferences into decision-making. As we envisage it, a deep learning system will support healthcare’s desire to continually improve, and make gains on the 60-30-10 dimensions. All modern health systems are awash with data, but it is only recently that we have been able to bring this together, operationalised, and turned into useful information by which to make more intelligent, timely decisions than in the past.

## A system in need of repair

Modern healthcare systems have a numbers problem: specifically, 60, 30, and 10. Despite all the resourcefulness and efforts of the past 30 years, the healthcare delivery cart remains stuck in a debilitating underperformance rut [1]. Care in-line with guidelines hovers at 60% as shown by large empirical studies of multiple conditions in adults and children in the USA, England, and Australia [2–6]. Some 30% of care is waste, duplication, or of low value, according to several authoritative sources including Berwick and the Organisation for Economic Co-operation and Development (OECD) [7–10], for which considerable expenditure cannot be justified. And many studies have documented how iatrogenic harm or adverse events befall at least 10% of patients globally [11–15].

Consider for a moment, if civil aviation, car manufacturing, or the software design industry achieved 60% reliability of service delivery in commercial passenger journeys, new motor vehicles, and just-released software programs. Imagine further if these sectors had a 30% inefficiency rate when producing their outcomes, and they harmed 1 in 10 of their customers. This would not, surely, be tolerated. Healthcare is more complex than those industries, but spends less effort on improvement.

These headline healthcare numbers persist and may become worse when we consider everything that is coming down the health innovation pipeline. Advances in precision medicine, genomics, new generation drugs, AI, and brain sciences are all in various stages of development or take up in healthcare—with the potential to do both good and harm to the system. If the 60-30-10 Challenge represents a strong signal that the system is not fit for purpose now, how will it cope with an avalanche of these advanced technologies? This new evidence has the potential to deliver new cures and to save and extend lives, but if not adopted effectively, or across-the-board, then the proportion of evidence-based care could fall, not rise. New technologies also increase the complexity of care—and add more risk, and if they do not provide an adequate return on investment may add more waste. They could also introduce more potential for increased iatrogenic harm. The 60-30-10 Challenge is standing in the way of progress.

## Where we are now?

To meet the Challenge, there is an urgent need for a conceptual leap in our understanding of how healthcare systems respond to relentless demands, internal and external pressures, and naturally evolve. Stretched clinicians and healthcare professionals see the symptoms of systems underperformance every day, so they are accustomed to frustrations, and, deft at work-arounds, mostly give of their best in a difficult system [16]. Managers, policymakers, ministers of health, and other politicians see it too, manifesting in the myriad of disparate matters (errors, human resource problems, politics, funding, and socio-economic issues) they have to grapple with, alongside weekly or daily media crises [17, 18]. It is not the workforce, any more than it is the patient, at fault. Today’s episodic, fragmented, and hierarchical models of healthcare delivery and organisational governance are straining because they are built for the past [15, 19, 20]. And the pace of change is exhausting, with people struggling to keep up. Indeed, new research is making around 7% of ‘best practice’ obsolete each year [21], and an average of five new diseases are added to Medline every week [22].

Meanwhile, research on healthcare systems, using increasingly sophisticated methods and approaches, and drawing on the same complexity science and network theories used to understand biological processes, is beginning to reveal deep insights into how things really work [23–26] (for definitions of terms, see Table 1). The answer is not the introduction of yet more rigid policies or re-arrangement of organisational charts in the vain attempt to restructure once again. That type of approach is based on linear thinking—to which humans all-too-often default. Such simplistic, if-then logic serves us well when making straightforward decisions, but it is insufficient for the wicked problems that now present to us in healthcare settings [29–31].

**Table 1:** Glossary of terms

Term	Definitions
Complex adaptive system	A dynamic, self-similar collectivity of interacting agents and their artefacts with emergent behaviours and characterised by nonlinearity, e.g. a large hospital.
Complexity	The behaviour embedded in highly composite systems or models of systems with large numbers of interacting components (e.g. agents, artefacts and groups); their ongoing, repeated interactions create local rules and rich, collective behaviours.
Complexity science	A discipline drawing on the study of systems sciences, accounting for and describing the core features and behaviours of different kinds of complex adaptive systems.
Emergence	Behaviours that are built from smaller or simpler entities, the characteristics or properties of which arise through the interactions of those smaller or simpler entities; the larger entities are one level up in scale and manifest as structures, patterns, properties, or collective behaviours.
Learning health system	A system at the crossroads of people and information systems—i.e. one that is ‘sociotechnical’—and that enables virtuous learning cycles through an underlying information infrastructure. Through the implementation of virtuous learning cycles, a learning system is informed by evidence and actionable data in ‘real-time’ and creates the foundations of a system capable of meeting systems-wide, clinically oriented, and patient-relevant delivery targets.
Network	An interlocking web of relationships or connections at varying levels of scale in a system; the agents or artefacts are the nodes and the relationships between them are lines or vectors, which together describe the structure of the interactions of the network’s membership.

Sources: Boeing [27]; Braithwaite et al. [24, 28]

**Fig. 1:**
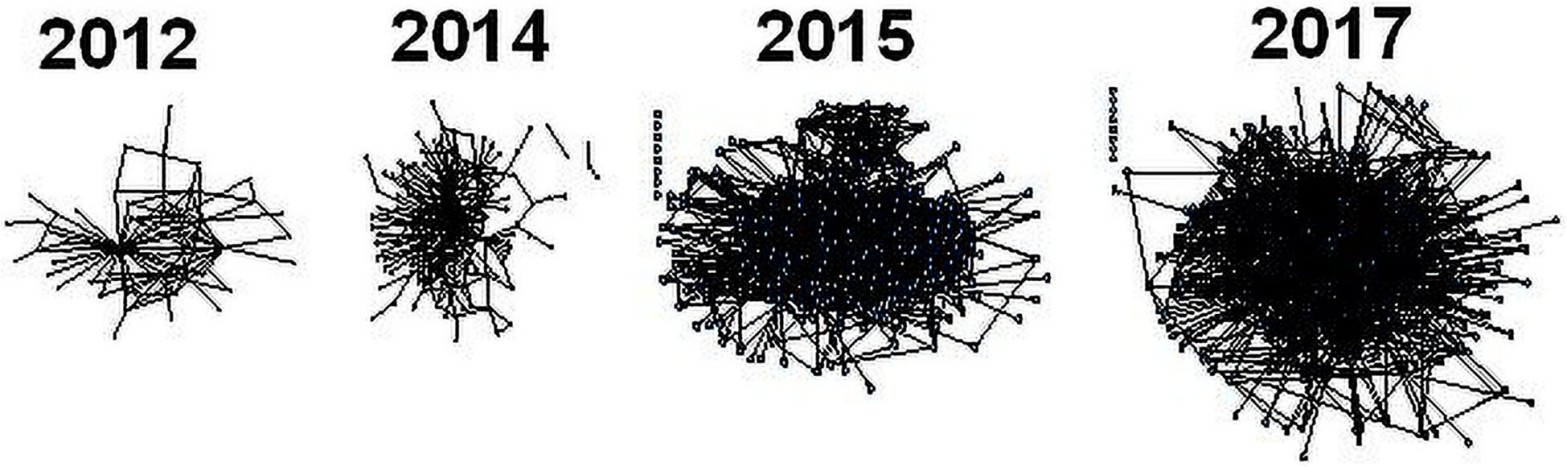
Social-professional network changes measured via a social network analysis of the Translational Cancer Research Network (TCRN) in Eastern Sydney, Australia. Each dot (node) represents a TCRN member, and each line (vector) a collaborative tie (adapted from Long et al. [33]). Permission is provided under Creative Commons Attribution License 4.0

Complexity science is making breakthroughs in understanding the dynamic webs of virtually infinite combinations of interactions required to deliver effective care. These complex healthcare ecosystems resist standardisation, and inevitably flex and adapt in the face of constant change and shifting pressures [32]. We can only improve them if we understand them as such. An example of how organic change across clinical practice can be induced is a network of clinicians, researchers, clinician-researchers, and patients in eastern Sydney, Australia, which nurtured their collaboration over a 6-year period, and achieved substantial growth in individuals involved in the collective strength of the network (Fig. 1). Network expansion was made possible through the allocation of research funding, so the partnership was strengthened diachronically. This included supporting the activities of opinion leaders and collaborators conducting and funding joint projects. An ethos of promoting inclusivity and teamwork was inculcated, and participation in educational and other events encouraged.

Managers might persist with hierarchical accountability charts and more policy pronouncements as solutions, but seeking to drive improvements to clinical practice via top-down edicts has rarely worked satisfactorily for those on the front lines of care. Complex healthcare systems do not respond in a linear way to what the high-level architects of change intend [28, 34]. But they do respond if provided with incentives, resources, encouragement, data, feedback, tools, and fewer constraints, as exhibited by the social network study. Across the 6 years, 2012 to 2017, network members could apply for funding together (incentives), a biobank was established (resources), members were supported by an administrative team to underpin progress and collaboration (encouragement, fewer constraints), and biostatistical support (data) and implementation science expertise (tools, feedback) were made widely available. Although direct attribution of the TCRN’s collaborative growth to downstream outcomes is difficult when so many variables in health systems are changing, there are some key accomplishments. The collaboration was funded by a modest research award of AUD$6.5 million in 2012 and again in 2017. In this time, the network published 1513 articles, leveraged a further $170,898,488 of funding, led or enabled over 50 research projects, and supported over 50 PhD students, and by February 2017, there were 2130 biobank participants. The TCRN initiated clinical improvement projects, including those to enhance quality of care in surgical oncology and multi-disciplinary care. In a 2015 survey of the network, a sub-sample of 122 respondents answering an open question indicated that changes in practice as a result of TCRN activities included enhanced engagement with consumers (42 respondents, 34%), the biobank (35, 29%), and diagnostic improvements around hereditary breast, ovarian, or colorectal cancer (5, 4%). By 2017, approximately two thirds of all respondents had been involved in new translational projects not funded by the TCRN but coming about as a result of TCRN involvement.

As well as beginning to understand the strengths of these kinds of networked collaborative structures, researchers have gradually realised that healthcare systems are non-deterministic and behaviours are emergent—that is, it is not possible to confidently predict the future by generalising from the past [28]. For example, medical errors have long been scrutinised using ‘root cause analysis’ that promises to identify and ‘fix’ the pathways to sometimes tragic failures. It is, of course, critical to establish how failures occur. But the answers may not effectively inform future safety protocols or avert further harm because few errors follow the same pathways within those complex webs of interactions that healthcare delivery entails. Initiating more rigid policies and procedures following such analyses in the expectation that the same confluence of events will arise in the future can have adverse effects by restricting the system and hampering the ability of teams to adapt in response to dynamic situations.

## A way forward

How then, can we use this knowledge and evidence from other examples [35–37] to break the impasse to achieve better, more cost-effective, and safer care? The 60-30-10 Challenge is ample warning against trudging along the same well-worn tracks to disappointment. Today’s popular ‘solutions’ such as restructuring [38], constantly fiddling with policy settings [39], adding more and more bureaucracy [40, 41], and introducing a new election manifesto or imposing fresh targets on the system every time a government changes [42] keep lots of people busy implementing ‘change’ but beyond superficialities; these measures conspire to constrain systems and contribute much inertia. In the end, all this top-down activity is not genuine improvement and just adds up to the same 60-30-10 gridlock.

Accumulating research across healthcare systems is reinforcing the view that we need to take the different approach that the network model signifies. We need to study, design, and test new integrated, interdisciplinary, and evidence-based models that can keep pace with inevitable changes in our knowledge, narrowing the gulf between research and clinical practice. These will be models that induce collaboration and transcend specialty silos; that link hospitals, primary care, aged care, and community services; and that can guide well-informed patients along clearer, evidence-based healthcare pathways, for their immediate health needs, and across their lifespan, from birth through paediatric to adult and aged care [23, 43, 44]. Such an approach requires multi-pronged strategies, from exploiting information and decision-support technologies to new health financing models that reward good care, de-fund futile or marginal care, and provide incentives to excel. That is just the first step.

We have been focusing intently on errors and waste while largely ignoring the majority of care that is delivered effectively, despite considerable pressures at the clinical coalface. Flip the question that way and we can ask how, in a system this complex, does so much care go well in everyday practice? [45] And, within the landscape of good care that is well delivered, where are the best examples of exemplary practice? Therein lie many of the secrets of success [26]. Learning from what goes right could help shift the dial on those headline numbers. In every healthcare system, we can dig a little deeper into each of those three figures to find useful variability. There are always stand out performers providing better care, creating less waste, or making fewer errors. They have much to offer other parts of the system operating under similar conditions.

Yet we are not very accomplished at spreading good practices across entire health systems, so islands of excellence can be found, but amongst oceans of poorer or even mediocre care, and the lessons are not shared, nor the better practices widely adopted. Those scattered exemplars already exist—such as the research networks for undiagnosed diseases [46] or the clinical improvement networks for cystic fibrosis [47], or more generally that better mortality and quality is correlated with clinical research, particularly the number of patients enrolled in interventional studies [48]—but they are not the norm, and even these examples can and must continue to reform and improve. In short, to understand health systems and systems performance, we need to focus not only on the problems (e.g. that harm or adverse events occur in 10% of admissions and GP encounters) but also on where things go well (e.g. where patients are kept safe in 90% of cases). For example, the WHO’s five moments for medication safety (starting, taking, adding, reviewing, and stopping a medication) [49] is an evidence-informed way of tackling errors (in the 10% camp), and Hollnagel’s resilience analysis grid (four resilience potentials: monitoring, learning, anticipating, and responding) [50] is a way of promoting more care going right (in the 90% camp).

Going further, there is potential to recalibrate static healthcare models so that organisations, clinical teams, and patients can learn for themselves, effectively improving processes on the go [51]. This is not fanciful and, in complex systems, is in reality the only way to proceed, because we simply cannot expect professionals on the front lines of care to respond to command and control management models rooted in the past. We have, or are assembling, the data mining tools, the ubiquitous digital connectivity, the mobile devices, and the burgeoning data banks and research registries to support coalface decision-making at virtually every step—and to constantly feed lessons back into care processes for continuous optimisation. This means harvesting big data, aggregated and configured as the engine of knowledge generation and application. It also means developing the next generation of clinicians such that they are adept with managing information and sure-footed with continuous improvement methods and systems-based approaches to practice [52]. Bring this together, and it is a learning system [53–55].

## The deep learning health system

**Fig. 2:**
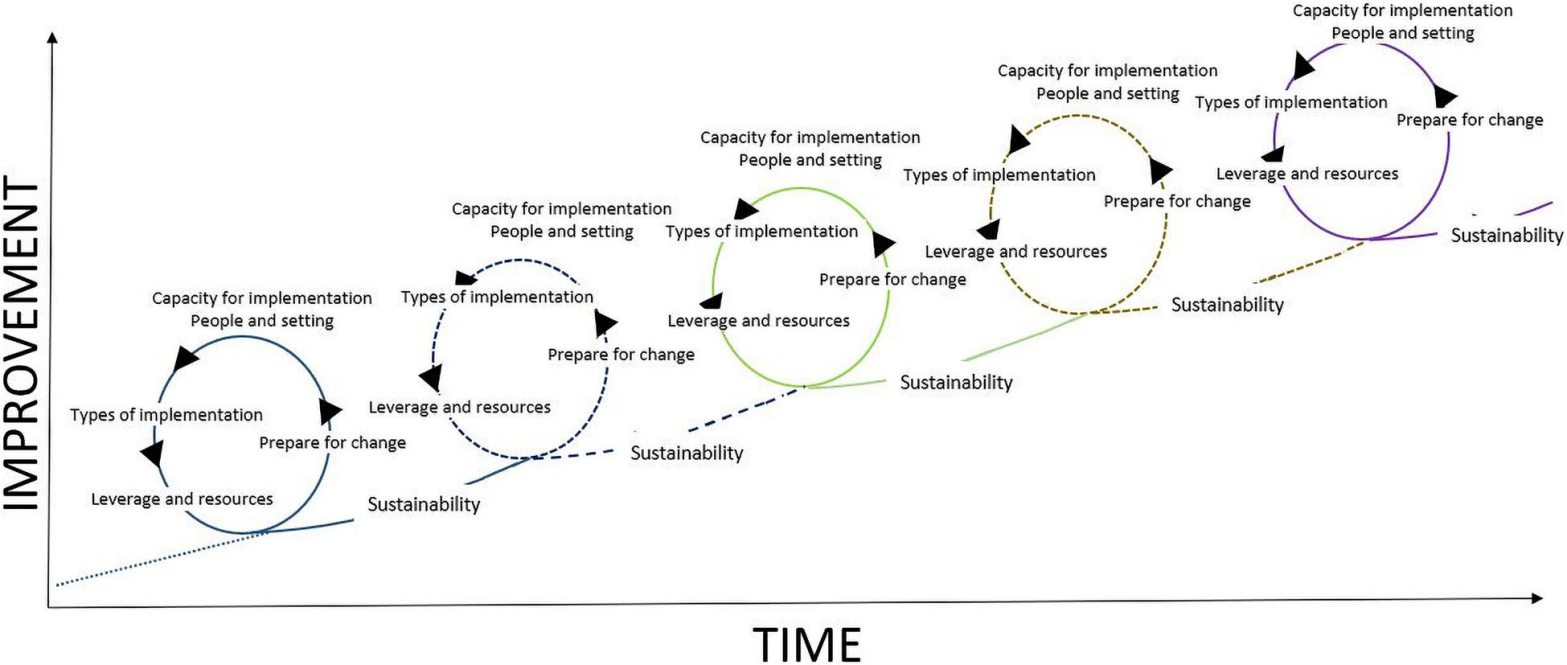
Phases of implementation as Formative Evaluation Feedback Loops (FEFL) (adapted from Braithwaite et al. [56] and Braithwaite et al. [57]). Used with permission from Oxford University Press

Schematically, the constantly improving system might behave something like the model in Fig. 2—where feedback is provided in close to real time to clinical teams, patients, managers, and policymakers, and efforts to improve care are much better aligned than today. The overarching idea is to measure progress with improvement over time in a learning environment [58] with a culture of respect and trust [59]. The drivers are as follows: being committed to improvement, a readiness and preparedness for change, recognising the capacities for and barriers to progress, an understanding of the types of implementation strategies available, and building sufficient leverage and allocating resources to the fast paced learning needed in today’s busy clinical world. While some medical school, nursing, and allied health programs have begun to emphasise the kinds of knowledge, competencies, and skills which will enable clinicians to be systems thinkers and change agents, working in adaptive learning systems and changing those systems as they go along, many do not—or they have not yet gone far enough. The US National Academies of Sciences, Engineering, and Medicine have argued recently that more people in healthcare need to be trained in systems thinking [15], underscoring our point.

**Fig. 3:**
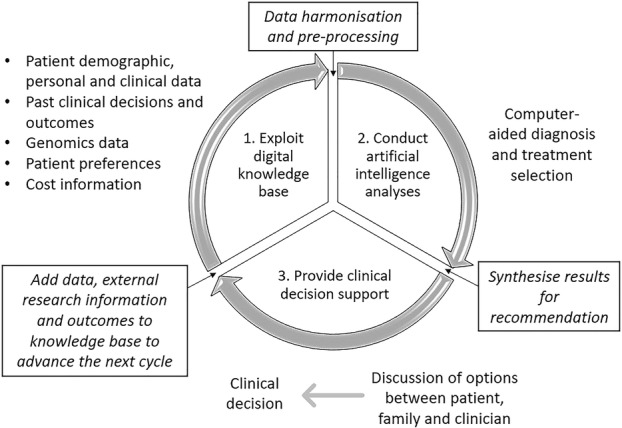
Cycles of advancement in the deep learning health system (adapted from Norgeot et al. [63]). Used with permission from Springer Nature

If we make progress in building rapid-learning systems predicated on ongoing improvement, regular feedback to stakeholders, and incorporating patients’ perspectives and choices into decisions [60], we can expect a variety of new models aligned with local conditions and workplace cultures to emerge, most likely centred on the clinical microsystem [61]. This is the defined, organised group of care staff and associated personnel looking after a targeted population of patients; a far cry from the outmoded concepts of care centred on the individual clinician on the one hand, or the top-down view of a highly structured and hierarchical system beloved of policymakers on the other. Our notion of a clinical-microsystem-as-learning-system is one that is adaptable and fluid rather than rigid and static—in other words, its features are much more closely aligned with the complex adaptive system of which it is an integral part. With such characteristics, the learning health system may be able to bring together and manage data from multiple sources, including information on health status, patients’ expectations and preferences, clinical and biological information, genomic data, cost and benefit schedules, and lifestyle and history profiles. All these data will, in the model we hope the system can aspire to, be brought together in useable forms for the benefit of both patients’ and clinicians’ decision-making [62]. If we get this right, such flexible and information-rich deep learning systems will replace today’s forgetting systems, with their entrenched, standardised, brittle organisational structures. Figure 3 shows how the cycles of advancement in the deep learning health system would work [63]. It is a high-level sketch outline of what might help us re-energise clinicians to provide more appropriate care, less waste, and safer, higher quality care—underpinned by the data needed to make good decisions, and adjust them over time.

Examples of such learning systems, or those attempting to emulate the characteristics of a learning health system, are emerging at a rapid pace. Cases in point are studies redesigning the care of lung cancer patients (The Ottawa Health Transformation Model) [64], the changing roles of researchers in different US settings using learning health system principles to reduce diagnostic errors and near misses [65], UK policy initiatives to build the infrastructure and data backbone on which the progress of learning health systems will be based [66], and data collaborations to reduce mortality associated with sepsis amongst 21 hospitals of the Kaiser Permanente North California system [67] following earlier Kaiser Permanente examples, such as the work on Vioxx, and the early detection of its long-term side effects [68].

## If we fail to make the transition

Despite being a relatively new idea, bringing together much that seems to be emerging and in-train in any case, such comprehensive systems models are not optional. We are acutely aware of the continued human and financial cost of current systems underperformance. All that poor care, waste, and iatrogenic harm cost billions of futile healthcare pounds, euros, and dollars. The very healthcare professionals we depend on to deliver quality care and to implement next-generation medical advances are labouring under unsustainable pressures and as a result, too often, feel they are failing their patients, or are burning out [69]. They need to know that the support mechanisms on which they rely are modernising, they have the tools to address the 60-30-10 Challenge, and they can deliver better care in a system that is daily becoming more complex.

Although the learning model is appealing, it is not guaranteed. In healthcare, some things are quickly accepted and embedded (e.g. laparoscopic techniques, immunisation of infants, day only surgery) and others have been slow in adoption (e.g. patient involvement in decision-making, various kinds of level 1 evidence, and adherence to guidelines such as for alcohol dependence, antibiotic use, and obesity) [5]. While there will be many variants on the theme, learning health systems will need to spread, principally through diffusion of innovation models and local adaptations [70]. Nevertheless, the combined thrust of thinking from the embryonic learning health systems literature [53–55], our TCRN case study and others we have pointed to, is attractive and does seem to represent a paradigm shift in re-conceptualising care.

## Conclusion

The learning health system model represents our best option at the moment for shifting the dial on these truculent numbers and rising to the Challenge. All-in-all, worldwide, we are investing heavily in biomedical and technological advances that promise safer, affordable, more effective healthcare. But without commensurate attention to fit-for-purpose, responsive, evidence-based delivery models that are built to learn and are commensurate with a complex systems view of healthcare rather than an inflexible, top-heavy, hierarchically laden command model, we will remain trapped in an Einsteinian Groundhog Day—doing the same thing over and over to achieve the same unsustainable results.

## Data Availability

Not applicable.

## References

[1] Braithwaite J. Changing how we think about healthcare improvement. BMJ. 2018;361:k2014. 10.1136/bmj.k2014 PMC5956926 29773537

[2] Braithwaite J, Hibbert PD, Jaffe A (2018). Quality of health care for children in Australia, 2012-2013. JAMA.

[3] Mangione-Smith R, DeCristofaro AH, Setodji CM, Keesey J, Klein DJ, Adams JL, Schuster MA, McGlynn EA (2007). The quality of ambulatory care delivered to children in the United States. N Engl J Med.

[4] McGlynn EA, Asch SM, Adams J, Keesey J, Hicks J, DeCristofaro A, Kerr EA (2003). The quality of health care delivered to adults in the United States. N Engl J Med.

[5] Runciman WB, Hunt TD, Hannaford NA, Hibbert PD, Westbrook JI, Coiera E, Day RO, Hindmarsh DM, McGlynn EA, Braithwaite J (2012). CareTrack: assessing the appropriateness of healthcare delivery in Australia. Med J Aust.

[6] Steel N, Bachmann M, Maisey S, Shekelle P, Breeze E, Marmot M, Melzer D (2008). Self reported receipt of care consistent with 32 quality indicators: national population survey of adults aged 50 or more in England. BMJ.

[7] Berwick DM, Hackbarth AD (2012). Eliminating waste in US health care. JAMA.

[8] OECD. Tackling wasteful spending on health. Paris: OECD Publishing; 2017.

[9] Saini V, Brownlee S, Elshaug AG, Glasziou P, Heath I (2017). Addressing overuse and underuse around the world. Lancet.

[10] Saini V, Garcia-Armesto S, Klemperer D, Paris V, Elshaug AG, Brownlee S, Ioannidis JPA, Fisher ES (2017). Drivers of poor medical care. Lancet.

[11] Baker GR, Norton PG, Flintoft V, Blais R, Brown A, Cox J, Etchells E, Ghali WA, Hébert P, Majumdar SR (2004). The Canadian Adverse Events Study: the incidence of adverse events among hospital patients in Canada. CMAJ.

[12] Brennan TA, Leape LL, Laird NM, Hebert L, Localio AR, Lawthers AG, Newhouse JP, Weiler PC, Hiatt HH (1991). Incidence of adverse events and negligence in hospitalized patients. N Engl J Med.

[13] Vincent C, Neale G, Woloshynowych M (2001). Adverse events in British hospitals: preliminary retrospective record review. BMJ.

[14] Wilson RM, Runciman WB, Gibbert RW, Harrison BT, Newby L, Hamilton JD (1995). The quality in Australian health care study. Med J Aust.

[15] National Academies of Sciences Engineering and Medicine, Health and Medicine Division, Board on Health Care Services, Board on Global Health, Committee on Improving the Quality of Health Care Globally (2018). Crossing the global quality chasm: improving health care worldwide.

[16] Braithwaite J, Hollnagel E, Hunte GS (2019). Resilient health care volume 5: working across boundaries.

[17] Li JW, Morway L, Velasquez A, Weingart SN, Stuver SO (2015). Perceptions of medical errors in cancer care: an analysis of how the news media describe sentinel events. J Patient Saf.

[18] Chester AN, Penno EC, Gauld RD (2018). A media content analysis of New Zealand’s district health board population-based funding formula. N Z Med J.

[19] World Health Organization, Organisation for Economic Co-Operation and Development, The World Bank. Delivering quality health services: a global imperative for Universal Health Coverage. Geneva: WHO, OECD, World Bank; 2018.

[20] Kruk ME, Gage AD, Arsenault C (2018). High-quality health systems in the Sustainable Development Goals era: time for revolution. The Lancet Global Health Commission on High Quality Health Systems in the SDG era.

[21] Shojania KG, Sampson M, Ansari MT, Ji J, Doucette S, Moher D (2007). How quickly do systematic reviews go out of date? A survival analysis. Ann Intern Med.

[22] What is a rare disease?. https://www.raredisease.org.uk/what-is-a-rare-disease/. Accessed 11 Mar 2020.

[23] Braithwaite J, Mannion R, Matsuyama Y, Shekelle P, Whittaker S, Al-Adawai S (2018). Health care systems: future predictions for global care.

[24] Braithwaite J, Churruca K, Long JC, Ellis LA, Herkes J (2018). When complexity science meets implementation science: a theoretical and empirical analysis of systems change. BMC Med.

[25] Reed JE, Howe C, Doyle C, Bell D (2018). Simple rules for evidence translation in complex systems: a qualitative study. BMC Med.

[26] Braithwaite J, Mannion R, Matsuyama Y, Shekelle P, Whittaker S, Al-Adawi S (2017). Health systems improvement across the globe: success stories from 60 countries.

[27] Boeing G (2016). Visual analysis of nonlinear dynamical systems: chaos, fractals, self-similarity and the limits of prediction. Systems.

[28] Braithwaite J, Churruca K, Ellis LA, Long J, Clay-Williams R, Damen N, Herkes J, Pomare C, Ludlow K (2017). Complexity science in healthcare – aspirations, approaches, applications and accomplishments: a white paper.

[29] Churchman CW (1967). Guest editorial: wicked problems. Manag Sci.

[30] Rittel HWJ, Webber MM. Dilemmas in a general theory of planning. Policy Sci. 1973;4(2):155–69.

[31] Cunningham FC, Ranmuthugala G, Westbrook JI, Braithwaite J (2019). Tackling the wicked problem of health networks: the design of an evaluation framework. BMJ Open.

[32] Hollnagel E, Braithwaite J, Wears RL (2018). Delivering resilient health care Abingdon.

[33] Long J (2016). Structuring successful collaboration: a longitudinal social network analysis of a translational research network. Implement Sci.

[34] Greenhalgh T, Papoutsi C (2018). Studying complexity in health services research: desperately seeking an overdue paradigm shift. BMC Med.

[35] Bhandari RP, Feinstein AB, Huestis SE, Krane EJ, Dunn AL, Cohen LL, Kao MC, Darnall BD, Mackey SC (2016). Pediatric-Collaborative Health Outcomes Information Registry (Peds-CHOIR): a learning health system to guide pediatric pain research and treatment. Pain.

[36] Finlayson SG, Levy M, Reddy S, Rubin DL (2016). Toward rapid learning in cancer treatment selection: an analytical engine for practice-based clinical data. J Biomed Inform.

[37] Barba P, Burns LJ, Litzow MR, Juckett MB, Komanduri KV, Lee SJ, Devlin SM, Costa LJ, Khan S, King A (2016). Success of an international learning health care system in hematopoietic cell transplantation: the American Society of Blood and Marrow Transplantation Clinical Case Forum. Biol Blood Marrow Transplant.

[38] Mannion R, Davies H, Marshall MN (2005). Cultures for performance in health care.

[39] Braithwaite J, Runciman WB, Merry AF (2009). Towards safer, better healthcare: harnessing the natural properties of complex sociotechnical systems. Qual Saf Health Care.

[40] Nugus P, Ranmuthugala G, Lamothe J, Greenfield D, Travaglia J, Kolne K, Kryluk J, Braithwaite J (2018). New ways to get policy into practice: a mixed-method participatory study of care coordination and street-level bureaucrats. J Health Organ Manag.

[41] Racko G (2017). Bureaucratization and medical professionals’ values: a cross-national analysis. Soc Sci Med.

[42] Mannion R, Braithwaite J (2017). False dawns and new horizons in patient safety research and practice. Int J Health Policy Manag.

[43] Amalberti R, Vincent C, Nicklin W, Braithwaite J (2018). Coping with more people with more illness. Part 1: the nature of the challenge and the implications for safety and quality. Int J Qual Health Care.

[44] Braithwaite J, Mannion R, Matsuyama Y, Shekelle PG, Whittaker S, Al-Adawi S, Ludlow K, James W, Ting HP, Herkes J (2018). The future of health systems to 2030: a roadmap for global progress and sustainability. Int J Qual Health Care.

[45] Braithwaite J, Wears RL, Hollnagel E (2017). Resilient health care volume 3: reconciling work-as-imagined and work-as-done.

[46] Splinter K, Adams DR, Bacino CA, Bellen HJ, Bernstein JA, Cheatle-Jarvela AM, Eng CM, Esteves C, Gahl WA, Hamid R (2018). Effect of genetic diagnosis on patients with previously undiagnosed disease. N Engl J Med.

[47] De Boeck K, Bulteel V, Fajac I (2016). Disease-specific clinical trials networks: the example of cystic fibrosis. Eur J Pediatr.

[48] Jonker L, Fisher SJ (2018). The correlation between National Health Service trusts’ clinical trial activity and both mortality rates and care quality commission ratings: a retrospective cross-sectional study. Public Health.

[49] World Health Organization, WHO Global Patient Safety Challenge (2019). 5 moments for medication safety. Medication Without Harm.

[50] Hollnagel E (2016). RAG - Resilience Analysis Grid.

[51] Burns J (2013). Are we on the way to a real ‘learning health care system’?. Manag Care.

[52] Wysham NG, Howie L, Patel K, Cameron CB, Samsa GP, Roe L, Abernethy AP, Zaas A (2016). Development and refinement of a learning health systems training program. EGEMS.

[53] Budrionis A, Bellika JG (2016). The learning healthcare system: where are we now? A systematic review. J Biomed Inform.

[54] Friedman C, Rubin J, Brown J, Buntin M, Corn M, Etheredge L, Gunter C, Musen M, Platt R, Stead W (2015). Toward a science of learning systems: a research agenda for the high-functioning Learning Health System. J Am Med Inform Assoc.

[55] Friedman CP, Allee NJ, Delaney BC, Flynn AJ, Silverstein JC, Sullivan K, Young KA (2017). The science of learning health systems: foundations for a new journal. Learning Health Syst.

[56] Braithwaite J, et al. Harnessing implementation science to improve care quality and patient safety: a systematic review of targeted literature. Int J Qual Health Care. 2014;26(3):321–9. 10.1093/intqhc/mzu047 24796491

[57] Braithwaite J (2007). An action research protocol to strengthen system-wide inter-professional learning and practice. BMC Health Serv Res.

[58] Jackson T (2014). Building the ‘continuous learning’ healthcare system. Health Inf Manag.

[59] Braithwaite J, Herkes J, Ludlow K, Testa L, Lamprell G. Association between organisational and workplace cultures, and patient outcomes: systematic review. BMJ Open. 2017;7(11):e017708. 10.1136/bmjopen-2017-017708 PMC5695304 29122796

[60] Slutsky JR (2007). Moving closer to a rapid-learning health care system. Health Aff.

[61] Mohr J, Batalden P, Barach P (2004). Integrating patient safety into the clinical microsystem. Qual Saf Health Care.

[62] Mohr JJ, Batalden PB (2002). Improving safety on the front lines: the role of clinical microsystems. Qual Saf Health Care.

[63] Norgeot B, Glicksberg BS, Butte AJ. A call for deep-learning healthcare. Nature Med. 2019;25(1):14–5. 10.1038/s41591-018-0320-3 30617337

[64] Fung-Kee-Fung M, Maziak DE, Pantarotto JR, Smylie J, Taylor L, Timlin T, Cacciotti T, Villeneuve PJ, Dennie C, Bornais C (2018). Regional process redesign of lung cancer care: a learning health system pilot project. Curr Oncol.

[65] Satterfield K, Rubin JC, Yang D, Friedman CP (2020). Understanding the roles of three academic communities in a prospective learning health ecosystem for diagnostic excellence. Learning Health Syst.

[66] Scobie S, Castle-Clarke S (2020). Implementing learning health systems in the UK NHS: policy actions to improve collaboration and transparency and support innovation and better use of analytics. Learning Health Syst.

[67] Liu VX, Morehouse JW, Baker JM, Greene JD, Kipnis P, Escobar GJ. Data that drive: closing the loop in the learning hospital system. J Hosp Med. 2016;11(Suppl 1):S11–S17. 10.1002/jhm.2651 PMC5510651 27805797

[68] Friedman CP, Wong AK, Blumenthal D (2010). Achieving a nationwide learning health system. Sci Transl Med.

[69] Lemaire JB, Wallace JE. Burnout among doctors. BMJ. 2017;358:j3360. 10.1136/bmj.j3360 28710272

[70] Greenhalgh T, Robert G, Macfarlane F, Bate P, Kyriakidou O (2004). Diffusion of innovations in service organizations: systematic review and recommendations. Milbank Q.

